# Inequalities in waiting times by socioeconomic status

**DOI:** 10.1186/2045-4015-3-38

**Published:** 2014-11-27

**Authors:** Luigi Siciliani

**Affiliations:** Department of Economics and Related Studies, University of York, New York, NY USA

## Abstract

Waiting times for elective care are a major health policy issue in many developed countries. Recent empirical studies suggest that inequalities in waiting times by socioeconomic status are present within publicly-funded systems in several countries. In this short commentary I discuss alternative approaches regarding data, methods and interpretation of results. Further work in this research area is needed.

## Introduction

Waiting times for elective (non-emergency) care are a major health policy issue in many developed countries. Average waiting times can reach several months for common procedures like cataract and hip replacement [1]. Waiting times are common in publicly-funded systems characterised by constraints on supply and limited use of prices.

Waiting times are unpopular with patients and the general public. They generate dissatisfaction for patients, since health benefits are postponed, patient’s health can deteriorate while waiting and suffering is prolonged. Governments have introduced a range of policies to reduce waiting times with varying degree of success [2].

Despite being unpopular, waiting times are often thought of as unavoidable but equitable within publicly-funded systems: patients with similar needs wait the same and access to care does not depend on ability to pay. This is unlike any form of price rationing, where poor patients may be deterred from demanding treatment if they find the price or co-pay expensive.

The study by Shmueli [3] points out that, unfortunately, patients’ waiting times are not based only on need in Israel. Richer patients wait less for a given level of need, even after controlling for the ability to pay privately. Some recent studies (reviewed in greater detail in Siciliani L: Waiting times: evidence of social inequalities of access to care. *Handbook Health Serv Res*. forthcoming) suggests that inequalities in waiting times by socioeconomic status are present in a number of countries, such as Australia, England, Italy, Norway, Spain and Sweden within publicly-funded systems [4–15]. Current studies differ in data sources and empirical approach.

In this short commentary I take the study by Shmueli [3] as an opportunity to discuss some of the alternative research approaches regarding data, methods and interpretation of results. My hope is that researchers will take up the challenge of further testing for inequalities in waiting times in the future.

### Data

#### What type of data can researchers employ?

Shmueli makes use of survey data for patients who had an MRI or (any) surgery. A nice feature of survey data is the accurate match of income/education variables with waiting times. The main disadvantage is that sample sizes tend to be small and the samples themselves tend to be relatively heterogeneous. Patients have different conditions, receive different treatments, differ in urgency and are admitted in different specialties. This may be problematic in at least two ways. Inequalities may be quite different across different treatments or specialties and therefore pooling may give a blurred picture. Information on treatments may be available, but adding too many control variables in an analysis with small sample size may make the estimation imprecise (i.e., not detecting a gradient when there is one).

I am aware of three other studies in this field that make use of survey data. Using data from SHARE, Siciliani and Verzulli [4] find evidence of inequalities in waiting times for publicly-funded specialist consultation and non-emergency surgery by socioeconomic status (education and income) for several European countries. Schoen et al. [5] use data from the 13th annual health policy survey conducted in 2010 by the Commonwealth Fund in eleven countries and report inequalities for some countries - mainly for waiting times for speciality consultation (see [6] for a separate study for Spain).

#### Is there an alternative to survey data?

Several countries measure waiting times as part of the regular data collection from administrative databases of the DRG type. Administrative data can potentially cover the whole population of patients admitted to hospitals for a specific treatment, therefore greatly expanding sample size and reducing heterogeneity. They have been usefully employed in analyses for Australia, England, Italy, Norway and Sweden [8–16] either for individual procedures (such as hip replacement and cataract) or all procedures. The main disadvantage of administrative data is the difficulty in linking patients’ waiting times with the patients' individual income or education (except for some Nordic countries), which is instead typically measured at the small-area level by matching patients with census data.

Both survey and administrative data pose different opportunities for controlling for the health status of the patient, its urgency and severity. Studies using survey data typically do so by controlling for self-reported health or the presence of chronic conditions. Administrative data typically include the number of registered comorbidities. An urgency gradient will remain even after excluding from the study population emergency patients with very short waiting times. It is therefore important to control for urgency and severity for elective patients since they can be correlated with income.

#### Which waiting-time measure?

As recognised by Shmueli [3], ideally we would like to measure the full duration of waits, once the surgery has taken place. I have called this in some previous work the “waiting time of patients treated” [1, 16], which can be available from both administrative and survey data. Some health systems record instead the “waiting time of patients on the list” at a point in time or a census date. By construction this measure is truncated and measures an incomplete wait. The distribution of waits of these two common measures are different but related [16–18] and both can be usefully employed.

## Methods

A range of regression approaches can be employed. Given the often discretised measure of waiting time in survey data (less than 1 month, 1–3 months, et) Shmueli [3] uses an ordered probit analysis. Siciliani and Verzulli [4] have a similar problem and use a negative binomial model (with data reported in 1-month or 1-week breaks). They suggest that the results are similar with an interval regression approach designed explicitly to deal with discretised data. With administrative data, where waiting times are measured in days, simple Ordinary Least Squares (OLS) models can do a good job at estimating inequalities by socioeconomic status [9, 10, 13, 14]. Alternatively, given that waiting is a duration variable, duration/survival models can be used, e.g. the Cox model [10]. Finally, we may be interested in identifying differences in waiting times at different points in the wait distribution: quantile-regression can serve for such purpose [7, 8].

Legitimately, Shmueli [3] is worried about sample selection. i.e. patients demanding treatment being different from those not demanding treatment, and such decisions are also dependent on income. This is one of several possible selection mechanisms. Sharma et al. [8] investigate inequalities in waiting times in Australia within publicly-funded hospitals. But in Australia about half or more of the patients obtain treatment in a private hospital, mainly paid through their private insurance. Since treatments are recorded both for public and private hospitals, Sharma et al. [8] estimate a Heckman selection model of the choice of opting for public versus private hospitals. Researchers may want to investigate other forms of selection mechanism depending on data availability and institutional setting.

### Interpretation

An obvious explanation for inequalities in waiting times by income in some health systems is the co-existence between public and private sectors, where patients have to pay out of pocket (or be insured) to access the latter. Patients who pay wait little in the private sector, and those who are not willing (or not able) to pay wait long for public treatment. But the literature seems to suggest that inequalities in waiting times are also present within the public sector where income or education should not matter.

What could generate inequalities within the public sector? I like to distinguish between inequalities “within” and “across” hospitals. Inequalities “across” public providers can arise if richer individuals live in wealthy neighbourhoods and hospitals located in such areas are better funded or managed, therefore offering shorter waits (whether this is the case depends on allocation formulas, shortages of particular types of surgeons, the prevalence of complex operations - which may make more difficult to schedule than routine ones, profitability of treatments, attractiveness of the areas for good doctors, etc.). Inequalities “within” a publicly-funded hospital arise if richer patients manage to get ahead in the queue (due to being more assertive, exploiting social networks etcs) or if poorer patients fall behind (e.g. miss appointments). The current literature suggests that inequalities in waiting times persist and are significant “within” publicly-funded hospitals [7–10, 13, 14].

## Conclusions

Inequalities in waiting times within publicly-funded systems by income or education have been identified in a number of countries including Australia, England, Italy, Norway, Sweden, Spain, and now Israel. As suggested by Shmueli [3] such results are “disturbing”. The result seems to be pervasive and to hold across different health systems [4–15]. More work is required to further understand the mechanisms causing such inequalities and to make a proper assessment of the phenomenon. There are a number of methodological, measurement problems and interpretation issues. I hope researchers will pick up the challenge.

## Author's information

Luigi Siciliani is Professor in the Department of Economics and Related Studies at the University of York. He is Director of the Graduate programme in Health Economics, and a Co-Editor of the Journal of Health Economics since 2008. He has worked extensively on waiting times in the health sector.

## Commentary on

Shmueli A: Do rich Israelis wait less for medical care? (2014) Israel Journal of Health Policy Research. 2014 Oct 30; 3:30.
